# Relationship of Amniotic Fluid Sludge and Short Cervix With a High Rate of Preterm Birth in Women After Cervical Cerclage

**DOI:** 10.1002/jum.15952

**Published:** 2022-02-02

**Authors:** Yingmin Huang, Xiaowen Liang, Jianyi Liao, Yingtao Li, Zhiyi Chen

**Affiliations:** ^1^ Department of Ultrasound Medicine The Third Affiliated Hospital of Guangzhou Medical University Guangzhou China; ^2^ Guangzhou Medical Centre for Critical Pregnant Women, Key Laboratory for Major Obstetric Disease of Guangdong Province The Third Affiliated Hospital of Guangzhou Medical University Guangzhou China; ^3^ Medical Imaging Centre, The First Affiliated Hospital, Medical Imaging Centre, Hengyang Medical School, University of South China Hengyang China; ^4^ Institute of Medical Imaging, University of South China Hengyang China

**Keywords:** amniotic fluid sludge, cervical cerclage, cervical length, preterm birth

## Abstract

**Objective:**

We aims to determine the relationship of amniotic fluid sludge (AFS) and/or short cervical length (CL, ≤25 mm) with a high rate of preterm birth in women after cervical cerclage.

**Methods:**

A retrospective cohort study was conducted among singleton pregnancies after cervical cerclage between January 2018 and December 2021. A total of 296 patients who underwent transvaginal ultrasound to evaluate CL and the presence of AFS within 2 weeks after cerclage were included. Pregnancy outcome after cerclage was analyzed in accordance with the presence of AFS and CL ≤25 mm.

**Results:**

In patients with cerclage, AFS was an independent risk factor for preterm birth at <28 and <36 weeks but not for preterm birth at <32 weeks, and CL ≤25 mm was an independent risk factor for preterm birth at <28, <32, and <36 weeks. The Kaplan–Meier analysis showed that the association between the presence of AFS and short gestational age at delivery was statistically significant in women with CL ≤25 mm (log rank test, *P* = .000). The Cox regression analysis showed that these results remained significant after adjusting for confounding factors (*P* = .000). The negative linear relationships between AFS and CL (*R* = −0.454, *P* < .001) also explained the outcome.

**Conclusions:**

AFS and short cervix have a direct effect on pregnancies after cerclage. Mid‐trimester AFS can become a supplementary ultrasound index for detecting preterm birth after cerclage in pregnant women with a short cervix.

As a serious pregnancy complication, cervical insufficiency contributes to adverse pregnancy outcomes, such as abortion or preterm delivery.^1^ Women diagnosed with cervical insufficiency benefit from cerclage placement to prolong gestation.^2^, ^3^, ^4^ However, several studies indicated that women still face the risk of preterm birth after cervical cerclage.^5^, ^6^, ^7^, ^8^, ^9^, ^10^, ^11^ Relevant guidelines regarding universal cervical length (CL) screening in low‐ or high‐risk women are available to identify women at risk for preterm birth, but the efficacy of continued CL surveillance for women after cerclage placement is controversial.^12^ A prior study showed that proximal CL is not linked to preterm birth in women with cerclage.^13^ A retrospective analysis also affirmed that CL after cerclage placement cannot predict latent labor.^14^ Identifying women at risk of preterm birth has been a longstanding goal. Therefore, characterizing the risk of preterm birth and selecting surveillance strategies after cervical cerclage are important. Amniotic fluid sludge (AFS), a kind of sonographic sign, is a free‐floating hyperechogenic material that develops close to the uterine cervix.^15^ AFS is an indicator of amniotic inflammation or infection.^16^ Moreover, the presence of AFS tends to have a high proportion in preterm birth.^17^ AFS is composed of a microbial biofilm, from which bacteria, such as genital mycoplasmas, *Ureaplasma urealyticum*, *Streptococcus mutans*, and *Mycoplasma hominis*, are previously isolated.^16^ Evidence indicated that the risk of preterm birth incurred by the presence of AFS is remarkably higher than that incurred by CL alone.^18^ However, relevant studies that predict preterm birth in women with a short cervix after cervical cerclage by using AFS are yet to be reported.

In this study, we have deeply explored whether the interaction between AFS and short cervix (CL ≤25 mm) influences preterm birth after cerclage. On the basis of this original study, we hope to determine an objective and efficient indicator of preterm birth for women after cerclage.

## Material and Methods

### Study Design and Subjects

A retrospective cohort study was conducted between January 2018 and December 2021 in The Third Affiliated Hospital of Guangzhou Medical University. The subjects were singleton pregnancies who underwent McDonald cerclage for the following diagnosis of cervical insufficiency based on a history of one or more mid‐trimester miscarriages or preterm birth (history‐indicated cerclage), transvaginal ultrasound evidence of short cervix (ultrasound‐indicated cerclage), and cervical dilatation found on speculum examination with amniotic sac bulging (rescue‐indicated cerclage).^1^ The exclusion criteria were (1) fetal congenital anomalies, (2) twin or multiple pregnancies, (3) obstetric complications (placenta previa, placental abruption, pregnancy‐induced hypertension, and gestational diabetes mellitus), (4) induction of labor at any gestational age, (5) incomplete clinical record, (6) abdominal cerclage/s, and (7) >1 cerclage suture placement (placement of cerclage in more than one occasion).

Perioperative antibiotic treatment was administered to women with cerclage to reduce the rate of complications during the procedure. However, different regimens, such as antibiotic type and treatment duration, were provided throughout the observation period. Cerclage suture was removed between 36 and 37 weeks or at the time when the patient developed progressing preterm birth. Pregnancy outcomes were monitored and recorded.

### Ultrasound Examinations

**Figure 1:**
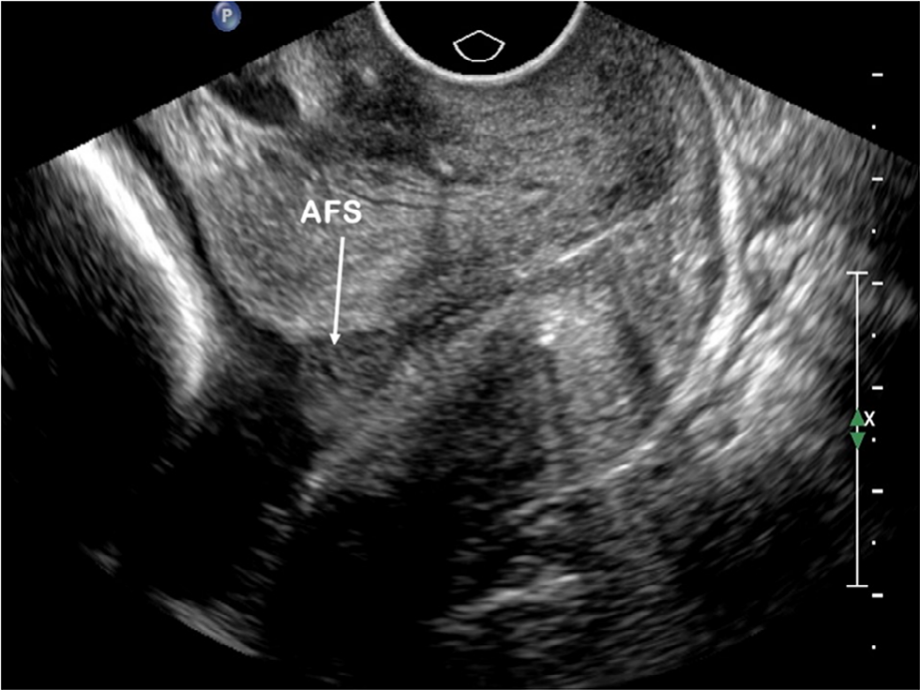
Sagittal view of the cervix showing AFS in a 32‐year‐old singleton pregnant woman at 20^+3^ weeks of gestation after cerclage placement. AFS (white arrow) is described as the presence of hyperechoic aggregates of particulate matter in the proximity of the internal cervical os.

All patients underwent transvaginal ultrasound at 14 to 24 weeks of gestation within 2 weeks after cerclage placement to measure CL and evaluate the presence of AFS. Ultrasound images were reassessed retrospectively by three ultrasound specialists who were blinded to pregnancy outcomes. Transvaginal ultrasound screening was conducted in accordance with the following standard technique.^19^ The patient was placed in dorsal lithotomy position after bladder emptying. A vaginal transducer (4–9 MHz) was inserted into the anterior fornix of the vagina to obtain a sagittal view of the cervix. The entire cervical canal could be visualized with no compression. CL was measured as the range from the internal cervical os to the external cervical os. The shortest of three CL measurements was recorded for further analysis. CL ≤25 mm was defined as a threshold for short CL. AFS was described as the presence of hyperechoic aggregates of particulate matter in the proximity of the internal cervical os (Figure 1). AFS could be broken into small particles by stimuli, such as fetal movement or manmade abdominal stimulation, but returned into clusters in minutes. A cluster with an average diameter >1 cm was defined as AFS(+).

### Ethical Approval

This study was approved by the Institutional Review Board of The Third Affiliated Hospital of Guangzhou Medical University (IRB no. 2021013), and all participants provided informed consent before enrolling in the study.

### Statistical Analysis

All data were analyzed using IBM SPSS Statistics 25. Categorical variables between the cases and control subjects were compared by Fisher's exact test or Pearson's chi‐square test. The Student's *t*‐test and Mann–Whitney test were used to assess continuous variables when appropriate. The interaction between AFS and CL was analyzed using the chi‐square test for trend and Pearson analyses. The multivariable logistic regression analysis was performed to adjust for confounding factors and predict preterm birth before 28, 32, and 36 weeks. The Kaplan–Meier survival analysis of gestational age at delivery was conducted in accordance with the presence of AFS in pregnant women with or without CL ≤25 mm. The Cox regression analysis was performed to assess the gestational age at delivery. Variables with *P* values <.05 in the univariate analysis were entered into multivariable logistic and Cox regression analyses. *P* < .05 was considered statistically significant.

## Results

### Maternal Characteristics

A total of 296 pregnant women were included in the analysis. The maternal characteristics of patients with and without AFS were compared as follows (Table 1). About 11.1% (33/296), 15.9% (47/296), and 28.0% (83/296) of patients gave birth before 28, 32, and 36 weeks, respectively.

**Table 1:** Characteristics in AFS(+) and AFS(−) Women

Characteristics	AFS(+)	AFS(−)	*P* value
No. of patients	45	251	—
Maternal age (year)	33.31 ± 4.92	33.12 ± 4.81	.807
Prepregnancy BMI (kg/m^2^)	24.63 ± 4.14	22.46 ± 3.56	<.001
Previous PTB	7 (15.6)	40 (15.9)	.949
GA at delivery (weeks)	31.76 ± 6.55	36.45 ± 4.33	<.001
Prior second‐trimester abortion	30 (66.7)	174 (69.3)	.723

Data are presented as mean ± SD and number (percent) where applicable; PTB, spontaneous preterm birth; BMI, body mass index; GA, gestational age.

### Percentage of Preterm Birth According to the Presence of AFS and Short Cervix (CL ≤25 mm)

**Figure 2:**
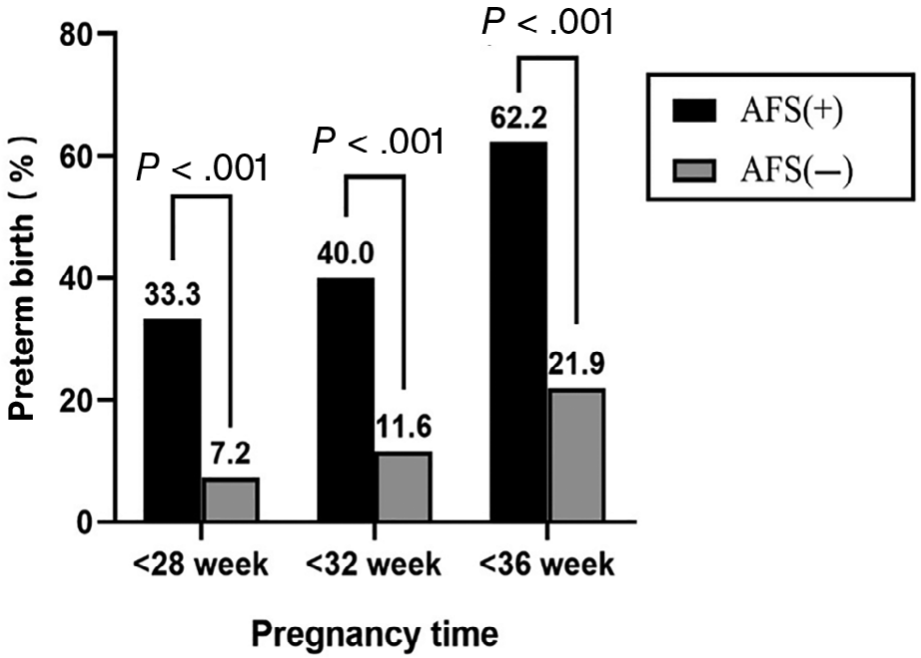
Percent of preterm birth at <28, <32, and <36 weeks of gestation in AFS(+) and AFS(−) patients.

**Figure 3:**
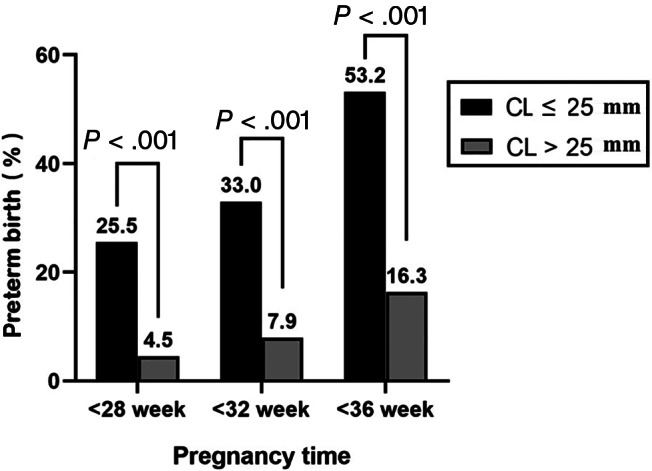
Percent of preterm birth at <28, <32, and <36 weeks of gestation in patients with CL ≤25 and >25 mm.

In this study, the prevalence of AFS was 15.0% (45/296), and the proportions of patients after cerclage with AFS that had preterm birth at <28 (33.3% [15/45] vs 7.2% [18/251], *P* < .001), <32 (40.0% [18/45] vs 11.6% [29/251], *P* < .001), and <36 (62.2% [28/45] vs 21.9% [55/251], *P* < .001) weeks were higher than those without AFS (Figure 2). Similarly, the prevalence of CL ≤25 mm was 32.0% (94/296), and the proportions of patients after cerclage with CL ≤25 mm that had preterm birth at <28 (25.5% [24/94] vs 4.5% [9/202], *P* < .001), <32 (33.0% [31/94] vs 7.9% [16/202], *P* < .001), and <36 (53.2% [50/94] vs 16.3% [33/202], *P* < .001) weeks were higher than those with CL >25 mm (Figure 3). The multivariable logistic regression analysis showed that in patients with cerclage, AFS was an independent risk factor for preterm birth at <28 and <36 weeks but not for preterm birth at <32 weeks, and CL ≤25 mm was an independent risk factor for preterm birth at <28, <32, and <36 weeks (Table 2).

**Table 2:** Multivariate Logistic Regression Analyses of AFS(+) and CL ≤25 mm Related to Preterm Birth After Cerclage

Preterm birth	AFS and CL	OR_obj_ (95% CI)	*P* value
At <28 weeks	AFS(+)	2.664 (1.096, 6.473)	.031
	CL ≤25 mm	4.392 (1.775, 10.864)	.001
At <32 weeks	AFS(+)	2.220 (0.988, 4.990)	.054
	CL ≤25 mm	3.766 (1.785, 7.944)	<.001
At <36 weeks	AFS(+)	2.522 (1.178, 5.398)	.017
	CL ≤25 mm	3.836 (2.081, 7.071)	<.001

### Detection of Pregnancy Outcome in Women With or Without CL ≤25 mm by Using AFS

**Figure 4:**
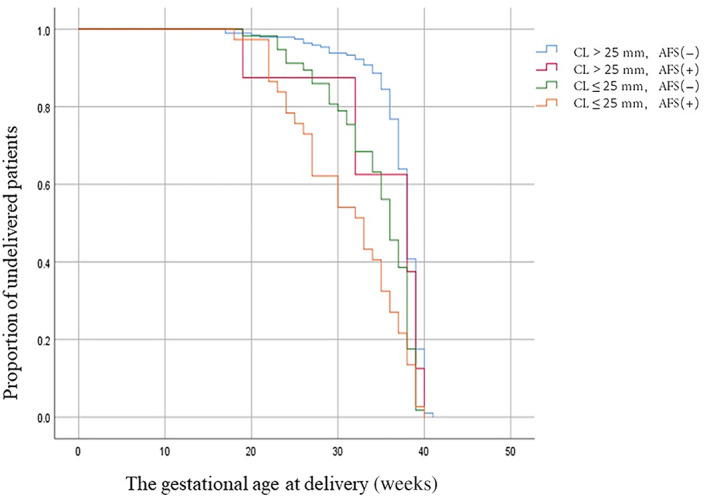
Kaplan–Meier analysis of the gestational age at delivery interval (weeks) in accordance with AFS and CL ≤25 mm results (log rank test, *P* = .000). Blue line, CL >25 mm, AFS(−); red line, CL >25 mm, AFS(+); green line, CL ≤25 mm, AFS(−); orange line, CL ≤25 mm, AFS(+). The above four groups have a median gestational age at delivery of 38, 38, 36, and 33 weeks, respectively.

The Kaplan–Meier analysis of gestational age at delivery (in weeks) was performed in accordance with AFS and/or CL results (Figure 4). The association between the presence of AFS and short gestational age at delivery was statistically significant in women with a short cervix (log rank test, *P* = .000). Specifically, patients with AFS(+) and CL ≤25 mm had shorter median gestational age at delivery than those with AFS(−) and CL ≤25 mm (33 vs 36 weeks). Patients with AFS(+) and CL ≤25 mm had a significantly shorter median gestational age at delivery than those with AFS(+) and CL >25 mm (33 vs 38 weeks). However, patients with AFS(+) and CL >25 mm had a same median gestational age at delivery with those with AFS(−) and CL >25 mm (38 vs 38 weeks). The Cox regression analysis showed that these results remained significant after adjusting for the presence of AFS, CL ≤25 mm, and prepregnancy body mass index (BMI) (hazard ratio, 2.45; 95% confidence interval, 1.7–3.5).

### Relationship Between AFS and CL

AFS and CL had a negative relationship (*P* < .001; Table 3). The Pearson analysis revealed a high rate of AFS in the presence of a short CL (*R* = −0.454, *P* < .001).

**Table 3:** Trend Change Between AFS and CL

Trend change of CL (mm)	AFS(+)	AFS(−)	*P* ^a^ value
CL ≥ 30	6 (3.3)	176 (96.7)	<.001
20 ≤ CL ≤ 29	13 (22.8)	44 (77.2)	
10 ≤ CL ≤ 19	18 (45.0)	22 (55.0)	
CL ≤ 9	8 (47.1)	9 (52.9)	

All data presented as number (percent).

^a^ The chi‐square test for trend: comparison of the trend change of CL between AFS(+) and AFS(−).

## Discussion

On the basis of previous studies, this study reports surveillance strategies to predict premature delivery in populations after cerclage. In this work, we discuss the relationship among AFS, CL, and preterm birth after cervical cerclage. Findings showed that the presence of AFS improves the specificity of a short cervix. The combination of AFS and CL ≤25 mm is an effective method for detecting preterm birth after cerclage.

AFS, an inflammatory exudate, is a component of fibrin, white blood cells, and germs.^20^ The present study has observed a high rate of AFS in the presence of short CL, which can deepen our understanding of the etiology of preterm birth. This finding is possibly because infection predisposes pregnant women to preterm birth through the activation of the mechanisms that lead to cervical ripening.^21^ However, the causal relationship by which AFS causes the cervix to shorten or predisposes women with an extremely short cervix to ascending infection remains uncertain. Nonetheless, AFS has received recognition as an effective index in treating preterm birth with antibiotics.^22^, ^23^

Several studies confirmed that cerclage has a positive effect on the prevention of preterm birth before 34 weeks of gestation.^24^, ^25^ In our study, the combination of AFS and CL ≤25 mm has a median gestational age at delivery of 33 weeks. Findings hint that the combination of AFS and CL ≤25 mm has an advantage in detecting preterm birth after cerclage especially in improving the identification of those who fail to benefit from cerclage. AFS improves the specificity of CL to a certain extent, which can avoid excessive intervention and reduce the previous controversy^12^ of whether CL should be applied successively in predicting preterm birth after cerclage.

The time of subsequent intervention is dependent on gestational age at surveillance evaluation, which is an obstacle to the survival of premature infants. In this study, the presence of mid‐trimester AFS and CL ≤25 mm after cerclage is closely associated with preterm birth. The above observation shows that all ultrasound indices make the most of their efficacy at the time of evaluation to avoid repeated ultrasound screening. This study has selected CL ≤25 mm as the binary classified variable to reduce false judgement^26^ so that the change trend of CL after cerclage cannot remain constant. Furthermore, we avoid an interference to AFS recognition, which is the presence of a large number of floating particles (vernix and meconium) in the amniotic fluid in the late gestational age.^27^

This study found yet another observation that the prevalence of AFS is relatively low, which indicates the instability of AFS. On the one hand, the floating particles, which represent bacteria, blood cells, vernix, and meconium, may be mistaken for AFS. On the other hand, a static image is difficult to analyze retrospectively. Moreover, the covering of the internal os by the fetal vertex, the presence of an active fetus, and change in maternal position leads to a subjectively false‐negative result (absence of AFS).^28^ However, from another perspective, some parts of AFS cannot be eliminated even with perioperative antibiotic treatment. Therefore, AFS can strongly predict preterm birth after cerclage.

In addition, other factors, such as BMI and cervical dilation, affect pregnancy outcomes after cerclage.^29^, ^30^ Notably, selecting an appropriate surveillance strategy is difficult because of the complexity of the mechanism of preterm birth. Variations in the study population limit the extensive popularization of surveillance strategies. The population of women with preterm birth after therapeutic intervention has variable and complex risk factors. For example, a study on pregnant women revealed that antibiotic treatment can reduce the number of AFS or even eliminate AFS in accordance with its initial size.^31^ Interestingly, our report strengthens the association of AFS and a short cervix to overcome the limitations of the low specificity and recognition ability of AFS and eliminate the confusion of past studies^32^, ^33^ that AFS is not suitable for detecting preterm birth after cerclage.

This study has some limitations. First, this study is a retrospective study and cannot estimate the exact natural frequency and history of occurrence of AFS. Second, a selection bias may arise because the decision of whether a cervical cerclage needs to be performed depends on the clinical criteria of the attending physician. The exact composition of AFS in this population is not confirmed in this study. Because of the lack of dynamic videos, we did not act a double‐check on the AFS cases.^34^ Finally, whether post‐cerclage CL rather than pre‐cerclage CL can effectively predict preterm birth after cerclage is uncertain. These limitations and further expectations of the present study are aims of another prospective studies.

## Conclusion

In conclusion, the combination of AFS and a short cervix improves the prediction of preterm birth in women after cervical cerclage. The clinical characteristics of AFS are not fully validated in clinical practice. The ultrasound features of AFS among patients presenting preterm labor need to be determined in further studies. AFS and a short cervix, which have implications for the management of preterm birth in women after cervical cerclage, can be used as biomarkers to detect preterm birth and offer clues to identify their mechanism in preterm birth.
